# Integrating telemedicine into nutritional management of infants with inherited metabolic disorders: a pilot study

**DOI:** 10.3389/fped.2026.1869508

**Published:** 2026-07-21

**Authors:** Veronica Maria Tagi, Chiara Montanari, Martina Tosi, Eliana Stucchi, Aurora Maria Esmeralda Cartalemi, Letizia Gandini, Francesca Eletti, Veronica Perico, Barbara Borsani, Laura Fiori, Valeria Calcaterra, Gianvincenzo Zuccotti

**Affiliations:** 1 Metabolic Diseases Unit, Department of Pediatrics, Vittore Buzzi Children’s Hospital, Milan, Italy; 2 Department of Biomedical and Clinical Sciences, University of Milan, Milan, Italy; 3 Department of Health Sciences, University of Milan, Milan, Italy; 4 Pediatric Unit, Department of Pediatrics, Vittore Buzzi Children’s Hospital, Milan, Italy; 5 Pediatric and Adolescent Unit, Department of Internal Medicine, University of Pavia, Pavia, Italy

**Keywords:** citrullinemia type I, growth, infants, inherited metabolic disorders (IMD), nutritional management, phenylketonuria (PKU), quality of life, telemedicine

## Abstract

**Background:**

Several inherited metabolic disorders (IMDs) require intensive and continuous nutritional management, particularly during the first year of life. Telemedicine (TM) may support care delivery in this setting, but evidence in infants remains limited.

**Objective:**

The aim of this pilot study was to assess the feasibility and acceptability of integrating TM into routine nutritional management of infants with IMDs identified through newborn screening.

**Methods:**

Caregivers of infants with IMDs requiring nutritional management were asked to choose between telemedicine-integrated follow-up (TM group) or exclusively in-person care (NO-TM group). Anthropometric parameters and clinical signs of malnutrition were assessed at birth, 6, and 12 months of life. All measured disease-specific biomarkers were collected. Caregivers assigned to the TM group completed structured questionnaires evaluating TM satisfaction and patients’ quality of life.

**Results:**

Nine infants with confirmed IMDs were enrolled and followed for 12 months. Seven patients (78%) were managed with TM integration. Growth trajectories and metabolic outcomes remained within expected clinical targets in both groups, and no clinical signs of malnutrition were observed in any patient. Disease-specific biomarkers in patients with phenylketonuria and citrullinemia type I remained within target ranges in both groups, with more stable phenylalanine levels observed in patients in the TM group. Caregivers reported high satisfaction with TM (median score: 94.5% of the maximum possible score). Quality of life scores were high in all patients.

**Conclusions:**

In this small pilot cohort, partial integration of TM into nutritional management of infants with IMDs appeared feasible, was well accepted by caregivers, and was not affected by evident adverse effects on growth or metabolic control during the first year of life. Larger studies are needed to confirm these observations and support wider implementation.

## 1 Introduction

Inherited metabolic disorders (IMDs) constitute a broad and complex group of rare genetic disorders characterized by a specific metabolic pathway dysfunction (1). Age at onset varies from the prenatal period to adulthood, with earlier onset generally correlating with worse outcomes. IMDs may manifest acutely, with metabolic decompensation, or chronically, with progressive multiorgan damage (2). Although individually these diseases are rare, the global prevalence of all IMDs is estimated to be about 5:10.000 live births, representing a considerable health burden given their high fatality (3). Indeed, it has been estimated that 23,529 child deaths from IMDs occur every year, accounting for 0.4% of all deaths in the paediatric age worldwide (3). The advent of newborn screening (NS) has revolutionized the clinical history of many of these diseases. In fact, early diagnosis enables timely therapeutic interventions which may prevent severe complications (4). Most diseases included in the national programs for NS in Europe and worldwide respond to a specific treatment. Nutritional intervention, including dietary restrictions, prescription of foods for special medical purposes (FSMPs) and micronutrient supplements, often represents the cornerstone of these patients’ management (1, 5). Caregivers are provided with specific guidance for the independent management of their children's diet; however, patients may require frequent adjustments based on disease marker values, growth, and clinical condition. Indeed, patients with IMDs at risk of metabolic decompensation, such as certain protein/ amino acid intoxication disorders, may need emergency dietary interventions during periods of increased protein catabolism, for example during intercurrent illness, in order to prevent metabolic crises (5).

The first year of life is a particularly critical period in the nutritional management of children with IMDs. Breastfeeding or bottle-feeding, as well as the introduction of complementary foods, require continuous adaptation according to the child's metabolic control and often involve the use of a wide range of FSMPs. In addition, caregivers must cope with a recent diagnosis and therefore need appropriate support to gradually achieve autonomy in managing their child's condition (5). It should also be noted that younger patients are at higher risk of urgent hospitalization in non-specialized hospitals, for example during an intercurrent illness, and in these cases a remote nutritional consultation may be needed by the healthcare personnel themselves.

In this context, telemedicine may represent a useful tool. The term ‘telemedicine’ (TM) refers to the provision of healthcare services through a remote electronic interface, either synchronously or asynchronously (6). Using information and communication technologies, it supports diagnosis, monitoring, treatment, prevention, and education within an integrated system of care (7). Teleconsultation is a specific telemedicine service that enables synchronous or asynchronous clinical interaction between patients and healthcare professionals, or among healthcare professionals, when participants are geographically separated (6). Several studies have demonstrated that, if teleconsultation is established with a proper communication and information exchange, good outcomes are obtained in terms of reduced travel and associated costs, decreased hospital admissions and length of stay, improved resource allocation, and strengthened coordination between care levels, with an overall enhancement of healthcare global quality (8, 9).

TM has proven to be an effective and cost-efficient solution also for the management of chronic and rare conditions, such as IMDs. Tertiary care centres are typically concentrated in urban areas, and these conditions often require multidisciplinary management by healthcare providers who may be located across different hospitals. This may limit access to care, leading to delays in therapeutic interventions and imposing a significant emotional burden on patients and caregivers (10). TM aims to address several key objectives in the management of rare and chronic diseases, ensuring continuity of care, frequent monitoring during application of emergency protocols, enhancing communication among healthcare professionals, supporting caregivers’ education, and, as a result, improving patients’ quality of life (11). In the field of IMDs, when integrated into standard care, TM may support rapid dietary adjustments, continuous assistance to caregivers, and coordination with non-specialized hospitals (12, 13).

The primary objective of this pilot study was to explore the feasibility and acceptability of integrating telemedicine into routine nutritional management during the first year of life in infants diagnosed with IMDs through newborn screening. A secondary exploratory objective was to descriptively evaluate growth patterns, metabolic control, caregivers’ satisfaction, and perceived quality of life among participants receiving telemedicine-supported care.

## 2 Methods

### 2.1 Study population and recruitment

The recruitment of study participants was carried out directly at the IMDs Reference Clinical Centre (RCC) of Vittore Buzzi Children's Hospital of Milan (Italy). The target population included all newborns referred to the RCC for a positive NS for an IMD, with confirmed diagnosis of IMD and requiring nutritional management, whose caregiver had access to an adequate internet connection and was in possession of a personal smartphone or computer. Patients with IMDs not identified through NS or with additional genetic diseases were excluded from the study. The panel of IMDs currently included in the National Program of Newborns Screening in Italy (14) is represented in Supplementary Figure 1.

Enrolment started in October 2023 and a 12-month follow-up was planned for all enrolled patients. Before enrolment, caregivers attended an in-person meeting with a paediatrician and dietitian of the RCC team, and received detailed study information. Interpreter support was available as needed.

### 2.2 Study design

Following the provision of written informed consent, caregivers were asked to choose whether to receive their nutritional consultations entirely in person (NO-TM group) or alternating in person appointments with telemedicine consultations (TM group), at a frequency based on the patient’s needs.

For all enrolled patients, we collected social and demographic data, all disease-specific biomarkers assessed during their first year of life. Furthermore, we collected anthropometric parameters at three predefined timepoints during an in-person visit: within the first 28 days of life (T0), at 6 months (T1), and at 12 months of age (T2). Auxological assessment included weight, length, and weight-for-length indices, expressed as Z-scores according to the World Health Organization (WHO) growth charts (15). Weight and height were measured using an electrical column scale (Soehnle Professional 7725, Soehnle Professional GmbH & Co. KG, Backnang, Germany) and an infantometer (Seca 416, Seca GmbH & Co. KG, Hamburg, Germany), respectively. In addition to the previously mentioned parameters, the possible presence of the following signs of malnutrition was assessed at T1 and T2, including low mid-upper arm circumference (MUAC) (16, 17) and reduced subcutaneous fat stores, evaluated by measuring triceps and subscapular skinfold thickness and comparing them with age- and sex-specific percentile distributions derived from National Health and Nutrition Examination Survey (NHANES) (18).

Caregivers of the TM group were also provided with an initial 30 min-training for the use of the TM platform at T0 and were administered two non-validated but structured questionnaires at T2: one to assess caregivers’ satisfaction with TM and one to assess patients’ quality of life. All social and demographic data records, TM platform-training, sample collection for disease-specific markers assessments, clinical evaluations at T0, T1 and T2 were performed in person at the Vittore Buzzi RCC for all participants.

Both satisfaction and quality-of-life questionnaires were administered verbally in person by a physician from the RCC at T2, and responses were recorded by the operator. An interpreter was present when needed.

**Figure 1:**
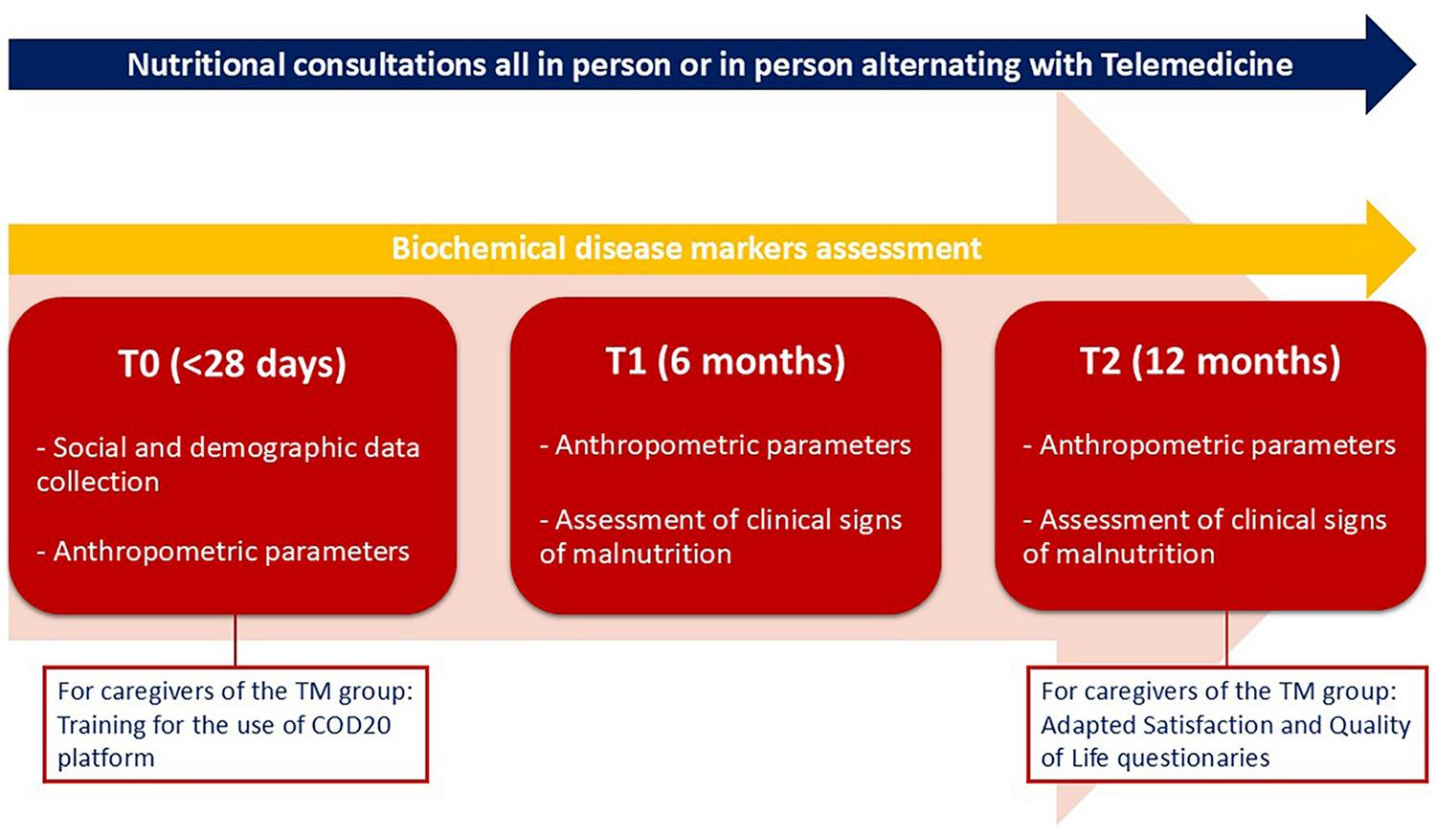
Simplified representation of the study timeline and assessments. IMD, inherited metabolic disorder; ID, identification number; MCADD, medium-chain acyl-CoA dehydrogenase deficiency; TM, received nutritional consultations through telemedicine; NO-TM, did not receive nutritional consultations through telemedicine.

Figure 1 provides a simplified overview of the study timeline.

#### 2.2.1 Nutritional management

Regardless of the mode of service delivery, all the nutritional evaluations involved a dietitian or nutritionist, in some occasions accompanied by a paediatrician of the RCC, and included the assessment of the child's diet through a 3-day food diary and analysis using the Metadieta software (MetadietaVR; METEDAsrl, via S. Pellico 4, San Benedetto del Tronto, AP, Italy), the evaluation of disease-specific markers and, when needed, nutritional indexes from blood tests, the planning or adjustment of a specific diet, when needed, according to the patient's age, growth and needs, and targeted supplementations based on the disease and blood test results.

TM consultations were performed through a digital telemedicine platform, named COD20, developed during the COVID−19 pandemic by the University of Milan in collaboration with the Health Protection Agency Metropolitan City of Milan, the Local Health and Welfare Agency Fatebenefratelli-Sacco hospitals, and industrial research partners (19). This platform provides a specialist video consultation service for patients that is fully integrated with the regional centralized appointment booking system.

#### 2.2.2 Satisfaction questionnaire

The satisfaction questionnaire (Supplementary Figure 2) was developed by adapting the Telehealth Usability Questionnaire (TUQ) originally proposed by Parmanto et al. (20), a validated instrument assessing usability and satisfaction with telehealth systems. The original questionnaire was selected as a methodological framework due to its established evaluation of key telemedicine domains, including ease of use, interaction quality, and overall satisfaction. However, the original questionnaire was designed for general telehealth applications and does not include domains specifically relevant to the care of infants with IMDs or to the burden experienced by caregivers managing complex dietary therapies. Therefore, the questionnaire was adapted to include additional context-specific domains regarding the nutritional management of children with IMDs and to add items related to perceived stress, economic burden, travel-related costs, and language barriers, which are not covered by the original TUQ.

The questionnaire is divided into three sections: in the first part (A) socio-demographical and logistical information about the caregiver was collected; in the second part (B) caregivers were asked to assign a score for each of the 16 items on a Likert 4-point scale from 1 (lowest satisfaction) to 4 (highest satisfaction), so that the total score ranged from 16 (lowest satisfaction) to 64 (highest satisfaction); in the third part (C), caregivers were asked to respond to five questions with yes/no answers.

Although the adapted tool was not formally re-validated in the study population, content validity was reviewed by the multidisciplinary clinical team (paediatricians and dietitians) to ensure clinical relevance and appropriateness for the target population.

#### 2.2.3 Quality-of-life questionnaire

The quality-of-life questionnaire (Supplementary Figure 3) was developed by adapting the PedsQL 4.0 Toddler Module [Varni et al. (21)], a widely used and validated instrument for assessing health-related quality of life in toddlers. However, several items in the original version are not developmentally appropriate for children at 12 months of age (e.g., running, lifting heavy objects, and active peer play). For this reason, the instrument was modified by removing items requiring developmental abilities not typically achieved at this age and by rephrasing selected items to improve interpretability for caregivers of infants. The resulting questionnaire therefore represents a shortened, study-specific version of the original PedsQL instrument, preserving its scoring structure but not its original domain structure. The primary purpose of these modifications was to ensure developmental suitability and content relevance rather than to preserve full comparability with the original validated scale. Given these modifications, the instrument was not re-validated, and results should be interpreted as exploratory and descriptive. In this questionnaire, the caregiver was asked to assign a score on a 5-point scale from 0 (never a problem) to 4 (almost always a problem) for each item assessing potential problems experienced by the child over the past month. Four items explored physical functioning, 4 items emotional functioning, and 3 additional items were proposed only to caregivers whose children attended nursery or day care in order to compare their capability of doing the same activities as their peers and ability to attend daycare depending on illness-related absences and hospital visits. Afterwards, each item was inversely converted into a number from 0 to 100, as follows: 0 = 100; 1 = 75; 2 = 50; 3 = 25; 4 = 0. Finally, the mean of all items for each explored area was calculated, to obtain a score ranging from 0 (lowest quality of life) to 100 (highest quality of life) for each functioning domain. Scores ≥75 were considered indicative of good quality of life.

### 2.3 Statistical analysis

Following completion of the one-year follow-up for all patients enrolled from October 2023 to April 2025, the cohort was divided into telemedicine users (TM) and non-users (NO-TM). Disease-specific biomarker levels were compared descriptively among patients with the same IMD for whom patients were available in both groups, while anthropometric parameters were compared across the entire cohort.

The questionnaires administered to caregivers of the TM group underwent textual analysis and graphical representation to describe the socio-demographic and logistical characteristics of participants, scores achieved for individual items and total scores.

Due to the small sample size, only descriptive statistics were performed. Continuous variables are presented as mean ± standard deviation, and categorical variables as counts and percentages. For individual Likert-scale items, mean values were calculated rather than medians to preserve data variability and avoid loss of information due to data aggregation. Data were visualized using line graphs, bar plots, and pie charts. No hypothesis testing was applied.

### 2.4 Ethics statement

This study was approved by the Local Ethics Committee LOMBARDIA 1 (Approval Committee name: Milano Area 1; approval no. Em. 81–2024, dated February 21st, 2024). All procedures adhered to the guidelines of the Local Ethical Committee and were performed in accordance with the ethical standards of the 1964 Helsinki Declaration. Informed consent for the treatment of anonymized data was obtained from a parent or a legal guardian. All collected data were pseudonymized and collected in a database established on the REDCap platform (https://www.project-redcap.org), hosted by Harvard Catalyst, Boston, USA, in strict compliance with applicable GDPR regulations.

## 3 Results

### 3.1 Study population

Nine patients (4 females and 5 males) were enrolled from October 2023 to April 2025: three with PKU (ID 1–3), two with citrullinemia type I (CTLN1) (ID 4 and 5), one with methylmalonic aciduria (MMA) (ID 6), one with *β*-ketothiolase deficiency (BKTD) (ID 7), one with medium-chain acyl-CoA dehydrogenase deficiency (MCADD) (ID 8), and one with hawkinsinuria (ID 9).

The 3 patients with PKU (ID 1–3), due to their elevated values of phenylalanine (Phe) at diagnosis (range 789–1676 µmol/L), required initial washout with suspension of breastfeeding or bottle feeding with regular infant formula and feeding with exclusive Phe-free infant formula for 24–48 h. Subsequently, natural proteins were reintroduced gradually, to achieve the maximum allowed Phe intake according to their individual tolerance, close monitoring of Phe levels on dried blood spot (DBS) and diet adjustments to reach the target range of 120–360 µmol/L according to European guidelines (22), supplementation with Phe-free or low-Phe protein substitutes (PS), special low-protein foods (SLPFs) and micronutrients when needed (23). Patient 1 was also affected by maternal PKU syndrome and was born with consequent microcephaly, low weight and short length.

The 2 patients with CTLN1 (ID 4 and 5) underwent nutritional consultations to assess the protein intake, verify an adequate caloric intake for age, introduce arginine supplementation and to establish an emergency dietary regimen according to guidelines (24).

Patient ID 6 was diagnosed with a mut0/mut- MMA responsive to cobalamin, however, in addition to parenteral hydroxocobalamin administration, this patient required a slight protein restriction diet during her first year of life to optimize the metabolic control (25).

For patient ID 7 a specific diet with restricted protein (1.5–2 g/kg) and controlled lipid intake was implemented, along with prevention of prolonged fasting according to the patient’s age and transient L-oral carnitine supplementation according to literature (26).

Patient ID 8 was diagnosed with MCADD with 0% enzyme residual activity. In his case, nutritional consultations were necessary to ensure that the child received an appropriate caloric intake and that the recommended age-specific fasting intervals were respected (27).

Hawkinsinuria is an IMD included in the differential diagnosis of non–type 1 tyrosinemia. Although patients’ prognosis is usually better that the latter, a low-tyrosine diet therapy may be required in some patients, along with ascorbic acid supplementation, which is recommended to support the activity of the 4-hydroxyphenylpyruvate dioxygenase enzyme, which is impaired in these patients (28). Patient ID 9 was identified through newborn screening with a suspicion of non–type 1 tyrosinemia, with initial tyrosine levels >500 µmol/L. Therefore, in addition to ascorbic acid supplementation, a low-protein diet and supplementation with a tyrosine- and phenylalanine-free infant formula were initially implemented for the first two months, followed by a gradual reintroduction of natural protein in accordance with the reduction in blood tyrosine levels according to recommendations (5).

Seven out of nine (78%) patients decided to take part in the TM group, while two decided to perform nutritional consultations exclusively in person (NO-TM group). The two patients who declined to use the TM service cited language barriers and the absence of a dedicated interpreter for remote consultations as reasons. One patient (ID 4) was lost to follow-up after transferring to another centre at 6 months of age.

Among the six patients who completed 12 months of follow-up in the TM group, telemedicine consultations alternated with in-person visits on a one-to-one basis. The frequency of consultations was individualized according to disease-specific requirements and patients’ clinical needs. The number of teleconsultations ranged from 6 to 18 per patient during the first year of life (median 12; IQR 6–18), with an equivalent number of in-person consultations performed for each participant. Patients’ characteristics and study group are summarized in Table 1.

**Table 1:** Characteristics of the 9 enrolled patients and participation group.

IMD	Total number of patients	Patient ID	Sex	Group
Phenylketonuria	3	1	M	NO-TM
		2	M	TM
		3	F	TM
Citrullinemia type 1	2	4	F	TM
		5	F	NO-TM
Methylmalonic aciduria	1	6	F	TM
Beta-ketothiolase deficiency	1	7	M	TM
MCADD	1	8	M	TM
Hawkinsinuria	1	9	M	TM

IMD, inherited metabolic disorder; ID, identification number; MCADD, medium-chain acyl-CoA dehydrogenase deficiency; TM, received nutritional consultations through telemedicine; NO-TM, did not receive nutritional consultations through telemedicine.

### 3.2 Patients’ growth

**Figure 2:**
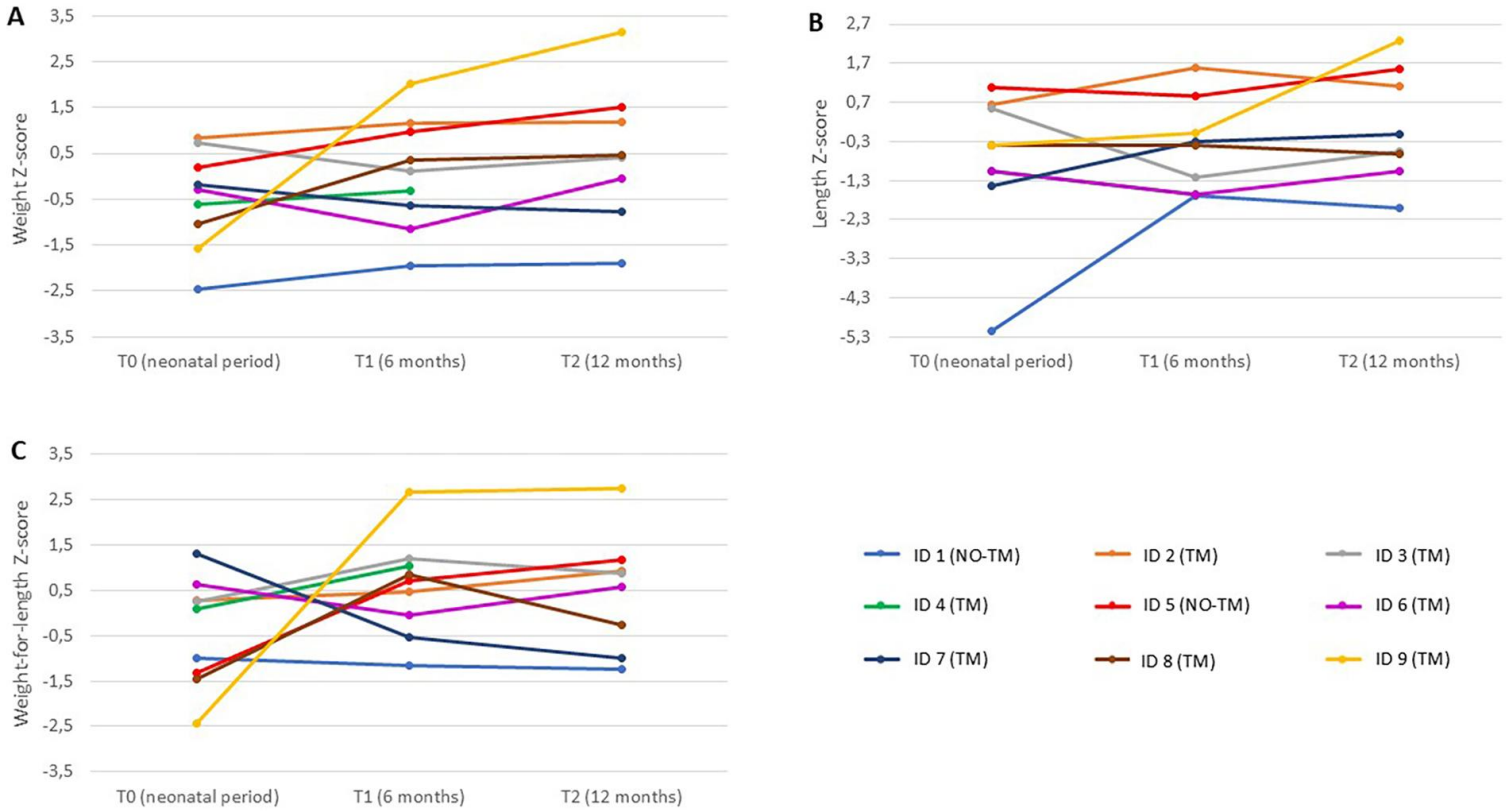
Anthropometric parameters Z-scores trends of each patient (ID 1-9) at T0 (neonatal period), T1 (6 months of age) and T2 (12 months of age) according to the world health organization growth charts (15). **(A)**: weight Z-score; **(B)**: length Z-score; **(C)**: weight-for-length Z-score. ID, identification number; TM, received nutritional consultations through telemedicine; NO-TM, did not receive nutritional consultations through telemedicine.

Trends of weight, length and weight-for-length Z-scores according to WHO charts at T0 (at birth), T1 (6 months) and T2 (12 months) were compared graphically for all patients in order to verify if growth was regular and to identify potential signs of malnutrition during the first year of life (Figure 2). Due to the drop-out, data at T2 are not available for patient ID 4.

Seven out of 9 patients (78%; ID 2–8) exhibited a regular growth of all the analysed anthropometric parameters, with Z-score values > −2 and < 2 at all time points. The remaining 2 patients (ID 1 and ID 9) showed marked deviations from WHO growth standards for reasons which are not directly related to their IMD. Patient 1 presented intrauterine growth restriction identified at 39 weeks of gestation, secondary to maternal PKU syndrome in a mother with unknown PKU and unmonitored pregnancy. He was born with microcephaly, low birth weight (Z-score: −2.45; Figure 2A) and short birth length (Z-score: −5.14; Figure 2B). However, during the following months, he showed a progressive increase of both weight Z-score (−1.94 at 6 months, −1.91 at 12 months) and length Z-scores (−1.7 at 6 months, −2 at 12 months) during the following months.

Patient 9 was born with low weight for gestational age (weight Z-score: −1.6; Figure 2A) and showed a rapid catch-up growth in the following months with adequate length and a weight-for-length Z-score of +2.7 at T1 and + 2.8 at T2 (Figures 2B,C). Caregivers were promptly provided with nutritional advice to avoid future overweight and consequent complications.

None of the patients exhibited low MUAC, reduced subcutaneous fat stores, or the presence of oedema at either T1 or T2 during follow-up.

In conclusion, except for two patients born small for gestational age and who underwent catch-up growth (one appertaining to the TM group and one to the NO-TM group), most patients showed a regular weight and length growth regardless of nutritional consultations delivery mode and no clinical signs of malnutrition were observed in any patient. Within the limitations of this small and heterogeneous cohort, no evident growth concerns emerged among patients receiving telemedicine-supported nutritional follow-up. Growth trajectories appeared generally consistent with expected patterns, although no formal comparison between groups was performed.

### 3.3 Patients’ metabolic control

#### 3.3.1 Phenylalanine values among patients with PKU

**Figure 3:**
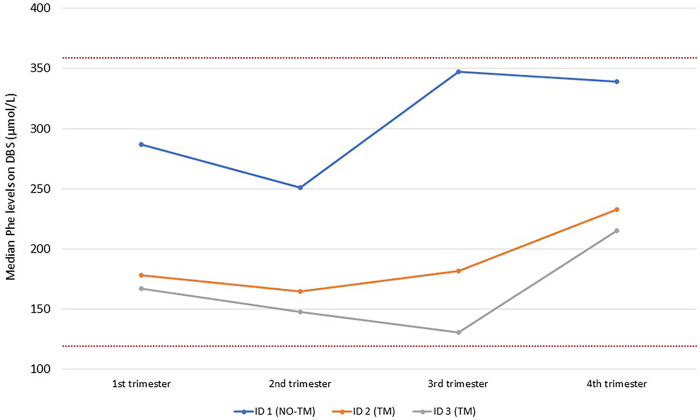
Median phenylalanine values trends on dried blood spot at each trimester of the three enrolled patients with phenylketonuria (ID 1-3) during their first year of life. The area within dashed lines represents the reference value range for age according to European guidelines (120-360 µmol/L) (22). Phe, phenylalanine; DBS, dried blood spot; ID, identification number; TM, received nutritional consultations through telemedicine; NO-TM, did not receive nutritional consultations through telemedicine.

Plasma Phe values, measured at least weekly on DBS, were collected during the first year of life of the three patients with PKU (ID 1–3). Trimestral median and standard deviations of Phe values were measured in order to assess each patient's metabolic control. We graphically compared the median Phe values at each trimester of patient number 1, who did not take advantage of telemedicine (NO-TM group) to patient number 2 and 3, who received nutritional teleconsultations (TM group), as shown in Figure 3. All median values of the three patients were included within the reference value range for age (120–360 µmol/L). However, regarding variability of Phe values, after a stabilization period in the first trimester, which occurred in all patients, patients 2 and 3 maintained much lower standard deviations than patient 1 (Table 2).

**Table 2:** Median phenylalanine values on dried blood spot and standard deviation at each trimester of the three enrolled patients with phenylketonuria (ID 1–3) during their first year of life.

ID	1st trimester		2nd trimester		3rd trimester		4th trimester	
	Median (µmol/L)	Standard deviation	Median (µmol/L)	Standard deviation	Median (µmol/L)	Standard deviation	Median (µmol/L)	Standard deviation
1 (NO-TM)	287	396	251	129	347	142	339	122
2(TM)	278	319	165	101	182	51	233	81
3(TM)	167	464	148	55	131	87	215	76

TM, received nutritional consultations through telemedicine; NO-TM, did not receive nutritional consultations through telemedicine.

In conclusion, among the patients receiving telemedicine-supported care, regular remote follow-up facilitated frequent dietary review and timely adjustment of nutritional prescriptions, maintaining a good metabolic control.

#### 3.3.2 Disease markers in patients with citrullinemia type I

Our study sample included two female patients with CTLN1, one included in the TM group (ID 4) and one taking part in the NO-TM group (ID 5).

**Figure 4:**
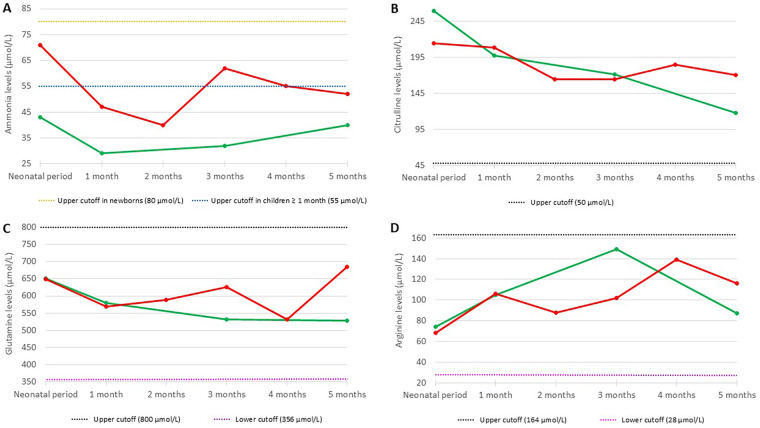
Ammonia **(A)**, citrulline **(B)**, glutamine **(C)** and arginine **(D)** plasma levels trends of the two enrolled patients with citrullinemia type 1 (ID 4 -green line, TM- and 5 -red line, NO-TM-) during their first 5 months of life. Dashed lines represent reference limits for age as detailed in the figure (24). ID, identification number; TM, received nutritional consultations through telemedicine; NO-TM, did not receive nutritional consultations through telemedicine.

Plasma levels of ammonia, citrulline, glutamine and arginine were collected as disease biomarkers to evaluate their metabolic control. Given the drop-out of patient 4 at 6 months, her biomarker values are available since the newborn age until five months, for this reason we graphically represented each biomarker value in this timeframe for both patients (Figure 4).

Ammonia levels remained below the cut-off for age (80 µmol/L in the neonatal age and 55 µmol/L afterwards) at all assessments in both patients (Figure 4A). Citrulline levels (Figure 4B) showed a similar trend in the two patients, with a slight reduction with the introduction of slight protein restriction and arginine supplementation and a stabilization of values higher than the cutoff for normal population (<50 µmol/L), but compatible with peers affected by the same IMD (>100 µmol/L).

Glutamine (Figure 4C) and arginine (Figure 4D) levels remained within the normal limits for age (glutamine: 356–800 µmol/L, arginine: 28–164 µmol/L). No arginine deficiency or accumulation was documented, suggesting a good compliance to the prescribed supplementation and its adjustments.

In conclusion, plasma disease biomarkers showed a good metabolic control in both patients included in the study, regardless of the mode of nutritional consultations delivery.

### 3.4 Caregivers’ satisfaction

The satisfaction questionnaire (Supplementary Figure 2) was administered to the caregivers of the 6 enrolled patients who received nutritional consultation through TM and whose child’s follow-up lasted at least 12 months (ID 2, 3, 6, 7, 8 and 9).

**Figure 5:**
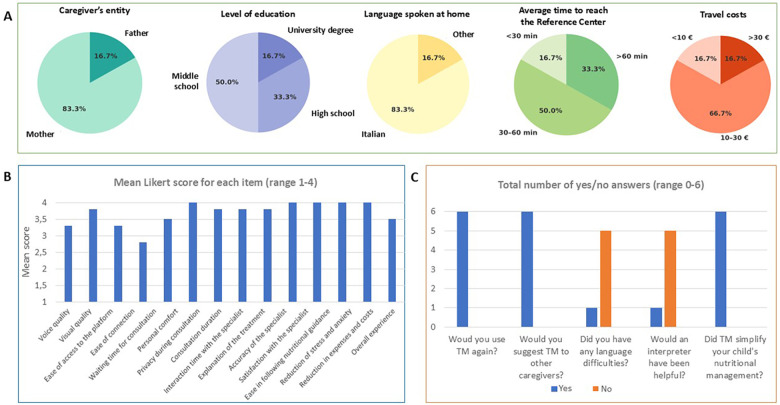
Results from the adapted questionnaire (Supplementary Figure 2) based on the telehealth satisfaction questionnaire adapted from parmanto et al. (20) for the 6 patients who received nutritional consultations through telemedicine and whose follow-up lasted at least 12 months (ID 2,3,6,7,8,9). **(A)**: socio-demographic characteristics of the caregiver who was administered the questionnaire, data regarding home distance from the Clinical Reference Centre and travel costs for each in person visit; **(B)**: mean Likert score for each item of the second part of the questionnaire on a 4-point scale from 1 (lowest satisfaction) to 4 (highest satisfaction); **(C)**: total number of yes/no answers to the five questions of the third part of the questionnaire. TM, telemedicine.

Figure 5 summarizes socio-demographic and logistic characteristics of the caregivers who were administered the questionnaire and the results of the questionnaire.

Five out of 6 caregivers who answered the questionnaire were the patients’ mothers (83%). Educational level was heterogeneous: 50% had completed high-school, two completed middle school and one had a university degree. For 5/6 patients’ families, Italian was the language primarily spoken at home, while for patient 8 both parents were Chinese native speakers.

Average time to reach the RCC from home was >60 min for 2/6 families (33%), between 30 min and 60 min for 3/6 families (50%) and <30 min for one (17%).

Travel costs to reach the hospital for each in person visit was estimated to range from 10 to 30 euros for 4/6 participants (67%), > 30 euros for one (17%) and <10 euros for one (17%) (Figure 5A).

The bar chart in Figure 5B shows the mean score achieved for each of the 16 items of the second part of the questionnaire (B) on a Likert 4-point scale (range: 1–4). The mean score for each item ranged between 2.8 and 4. Maximum scores were attributed to 6 items, related to personal comfort, ability to interact with the healthcare personnel, clarity of treatment explanations, satisfaction with the healthcare providers and cost reduction. The lowest scores were attributed to connection adequacy and ease of access to the platform. Total scores variability was minimal among participants, ranging from 54 to 63, with a median of 60.5/64 (interquartile range, IQR: 58.25–62), corresponding to 94.5% of the maximum score, and a mean of 59.7 (standard deviation, SD: 3.4).

The bar chart in Figure 5C shows the number of positive and negative answers given by each caregiver to the 5 yes/no questions asked in the third part of the satisfaction questionnaire (C). All participants stated that they would use TM again and would recommend it to other caregivers. Moreover, all caregivers acknowledged that the use of TM simplified their children's routine nutritional management.

The only non-Italian native-speaking caregiver reported difficulties linked to the language barriers and stated that the presence of an interpreter would have improved the experience. Importantly, two families who declined participation in the TM group cited language barriers and the lack of dedicated interpreter support during remote consultations as the main reasons for preferring exclusively in-person visits.

Overall, TM users expressed a favourable opinion about the use of this tool in the nutritional management of their children, and the main reported disadvantage was a communication issue secondary to language barriers, which could be overcome with the implementation of the service with a dedicated interpreter.

### 3.5 Patients’ quality of life

The quality-of-life questionnaire (Supplementary Figure 3) was administered to the caregivers of the 6 enrolled patients who received nutritional consultation through telemedicine and whose child’s follow-up up lasted at least 12 months (ID 2, 3, 6, 7, 8 and 9).

**Figure 6:**
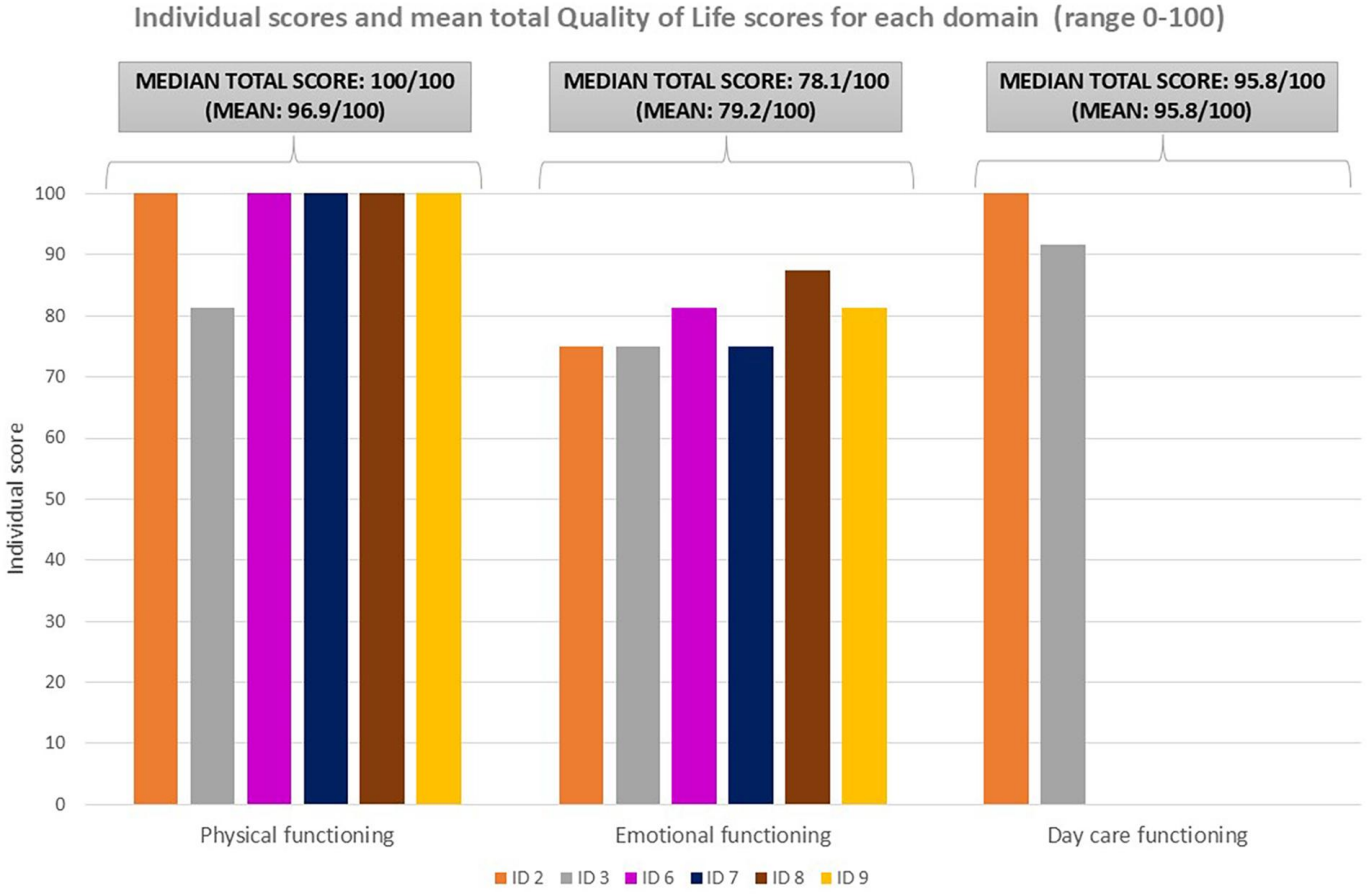
Results from the quality-of-life questionnaire (Supplementary Figure 3) adapted from the PedsQL 4.0 for toddlers (21) for the 6 patients who received nutritional consultations through telemedicine and whose follow-up lasted at least 12 months (ID 2,3,6,7,8,9). The bar chart represents individual scores, total median and mean scores of all patients for each domain explored: physical functioning, emotional functioning and day care functioning when applicable (ID 2 and 3).

Figure 6 summarizes the results of the questionnaire.

In the physical functioning domain, patients’ scores ranged from 81.3/100 to 100/100, with a median of 100 (IQR: 100–100) and a mean of 96.9/100 (SD: 7.7).

In the emotional functioning domain, scores varied between 75/100 and 87.5/100, with a median of 78.1 (IQR: 75–81.3) and a mean of 79.2/100 (SD: 5.1).

The day care functioning domain was assessed only in two patients (ID 2 and ID 3), as the remaining participants did not attend day care. In this subgroup, scores were 100/100 and 91.7/100 respectively, with a mean of 95.8/100 (SD: 5.9).

In conclusion, the questionnaire revealed generally favourable caregiver-reported quality of life scores among participants in the telemedicine group, with both physical and emotional well-functioning reported after a one-year follow-up. Similarly, very high quality of life scores were observed in this subscale among the two children attending day care.

## 4 Discussion

This pilot study provides preliminary real-world data on the feasibility of integrating TM into the nutritional management of infants with IMDs during the first year of life. Furthermore, to the best of our knowledge, this is the first study to explore growth and nutritional status in patients with IMDs receiving a nutritional management integrated with TM.

In our cohort, TM-supported follow-up appeared compatible with adequate growth. Indeed, following adequate nutritional follow-up, none of the patients showed clinical signs of malnutrition at 6 and 12 months. Growth trajectories remained within expected clinical targets in both TM and NO-TM groups in patients with the same IMD, with no evidence of impaired growth in patients managed through a combination of in-person and TM consultations. Alternating between in-person visits and TM follow-ups did not appear to have a negative impact on growth outcomes in our cohort. However, further studies with larger cohorts are warranted to confirm these preliminary results.

Metabolic control was assessed in the two diseases for which NO-TM controls were available: PKU and CTLN1.

Regarding patients with PKU, all three patients showed median Phe levels within the normal range at each trimester. The two patients receiving telemedicine-supported care appeared to show lower variability, expressed as standard deviation, in Phe values after the stabilization period; however, given the very small sample size, no conclusions regarding the effect of telemedicine can be drawn. However, our findings are consistent with the existing literature: a study from Zubarioglu et al. (29) suggests that TM may improve plasma Phe control in patients with PKU thanks to more frequent contact with the RCC.

The two patients with CTLN1 included in our cohort showed ammonia levels always within reference ranges throughout the observation period, regardless of their study group. Similar results were obtained also in glutamine levels, another relevant marker of metabolic control, being closely related to ammonia values. Arginine levels also showed a similar pattern in both patients, with moderate fluctuations but consistently remaining within normal ranges. This finding confirms appropriate arginine supplementation and is consistent with guidelines, stating that moderate fluctuations in plasma concentration are physiological and supplementation should be adjusted over time according to the patient's clinical and biochemical status (24). No episodes of metabolic decompensation occurred in either patient during the observation period, and neither hospitalization nor treatment with nitrogen scavengers was required. These observations in the two patients’ disease markers suggests that, when properly structured, TM may serve as a promittent complementary tool to ensure good metabolic control in patients with CTLN1, particularly during the first year of life. However, given that only two patients with CTLN1 were included, one of whom was lost to follow-up, these observations should be interpreted with extreme caution. Nevertheless, the use of TM has been investigated in several clinical settings, where it has demonstrated non-inferiority compared to in-person care in terms of patients’ clinical outcomes (30, 31).

Approximately half of the families assigned to the TM group reported a significant economic and time burden associated with travel to the RCC, highlighting how TM addresses a real need for logistical and economic simplification, particularly benefiting families living at greater distances or in more vulnerable conditions. These data suggest that TM may address family needs, related not only to the distance from the hospital, but also for time-managing and costs.

Our audit revealed an extremely positive perception of telemedicine as an adjunctive tool to clinical practice. Likert scale scores indicated strong satisfaction regarding communication with physicians, clarity of explanations, perceived professionalism, and personal comfort. Slightly lower scores reflected technical issues that, however, did not appear to negatively affect the overall experience. Furthermore, non-native speaking families may face additional barriers to accessing TM services; therefore, implementation of language mediation should be considered.

The overall total scores suggest an extremely positive and consistent perception among participants and the technical and language issues reported by some caregivers represent an area of improvement but did not appear to have compromised the overall quality of the experience. These findings are consistent with previous studies in other fields of paediatrics, where TM has been associated with high levels of users’ satisfaction. A previous study conducted on hospitalized children supported by the same TM platform, demonstrated efficient performance in early-discharged patients and high levels of caregivers’ satisfaction (32). A study in paediatric neurology confirmed high satisfaction among families and caregivers, supporting the validity of telemedicine even in complex clinical settings (33). In the study by Rovelli et al. (34) TM achieved high satisfaction also among caregivers of children with PKU, especially of those younger than 3 years.

In the third section, all caregivers expressed willingness to reuse the service in the future and recommend it to others, recognizing its role in simplifying disease management. This unanimous agreement represented an important indicator of acceptability and sustainability of the model.

As reported in the literature, barriers to in-person care can be mitigated through TM, particularly for children with complex conditions; it can bridge gaps in access to care, especially for families in rural areas or with limited transport options, particularly when patient mobility is impaired (35). TM interventions reduce geographical and temporal barriers, creating a bridge between families’ homes and tertiary care centres, improving equitable access to care, reducing work and school absences, and lowering healthcare and travel costs (36–38).

However, it should be noticed that TM should not replace clinical practice, not only because in-person visits allow for a range of assessments that are not feasible through a screen, but also because it cannot replicate the interpersonal relationships established during face-to-face consultations, as highlighted by McBride et al. (39). Furthermore, the reported issue of language barriers by a caregiver suggests the need to strengthen intercultural support, for example through the integration of professional interpreters.

Regarding the adapted quality of life questionnaire, the questionnaire revealed generally favourable caregiver-reported quality-of-life scores among participants in the telemedicine group. This finding, consistent with the literature, supports the hypothesis that infants’ adequate psychophysical well-being can be maintained even in remote care settings (40, 41).

The present study has several limitations that should be considered in these findings’ interpretation. First, the sample size was small, reflecting the rarity of IMDs, and the study groups were unbalanced, with substantially more participants in the TM group than in the NO-TM group. In addition, one patient in the TM group was lost to follow-up during the study period. Together, these factors limited the possibility of group comparisons and precluded the use of inferential statistical analyses.

A further limitation is the clinical heterogeneity of the cohort. Patients with six different IMDs were enrolled, each characterized by distinct disease severity, nutritional requirements, metabolic targets, and follow-up needs. Consequently, disease-specific conclusions cannot be drawn, and the generalizability of the findings should be interpreted with caution.

Another important limitation concerns the self-selection of participants into study groups. Caregivers were allowed to choose whether to participate in TM-supported care, introducing the possibility that families opting for TM differed from those choosing exclusively in-person care in terms of digital skills, health literacy, motivation, socioeconomic characteristics, logistical constraints, or confidence in disease management. These factors may limit the ability to attribute the observed findings directly to the TM intervention.

Our findings also highlight a potential equity issue: families requiring language mediation may face additional barriers to accessing TM services and could therefore be at increased risk of healthcare inequities if adequate support is not available. Therefore, future studies should evaluate TM models incorporating professional interpreter services in order to reduce the risk of health inequities.

In addition, results from the caregiver satisfaction and quality-of-life questionnaires should be interpreted cautiously, as both were adapted from previously validated instruments to tailor them to the context of nutrition in infants with IMDs, without formal re-validation. Therefore, the psychometric properties of the modified questionnaires cannot be assumed and may limit comparability with other studies using the original validated tools. For this reason, our findings should be interpreted as exploratory rather than confirmatory. Moreover, quality-of-life data were collected only in the TM group; therefore, no conclusions can be drawn regarding the impact of TM on quality of life compared with standard care.

Finally, the follow-up period was limited to the first year of life and does not allow conclusions regarding long-term outcomes.

Overall, given the small sample size, non-randomized design, and clinical heterogeneity of the cohort, these findings should be interpreted with caution and considered exploratory. Nevertheless, this pilot study suggests that partial integration of TM into routine nutritional management of infants with IMDs is feasible and well-accepted, particularly among patients with PKU and CTLN1. However, the study was not designed nor powered to evaluate effectiveness or equivalence compared with conventional care. Larger prospective studies with longer follow-up periods, more homogeneous patient populations, validated assessment tools, and more balanced comparison groups are needed to further explore the potential role of TM in the nutritional management of IMDs across different ages.

## 5 Conclusion

This pilot study suggests that integrating telemedicine into the nutritional management of infants with inherited metabolic disorders is feasible and well accepted by caregivers. Telemedicine-supported follow-up appeared compatible with appropriate growth monitoring and metabolic management in this small cohort; however, no conclusions regarding effectiveness or equivalence with standard care can be drawn. Larger prospective studies with more homogeneous populations and peer-matched controls are needed to further evaluate the efficacy and safety of telemedicine in the nutritional management of children with inherited metabolic disorders.

## Data Availability

The datasets presented in this article are not readily available because of privacy and ethical restrictions but are available upon reasonable request. Requests to access the datasets should be directed to chiara.montanari@unimi.it.

## Supplementary material

Supplementary Figure 1 Panel of IMDs currently included in the National Program of Newborns Screening in Italy (14).

Supplementary Figure 2 Adapted questionnaire based on the telehealth satisfaction questionnaire developed by Parmanto et al. (20). The questionnaire is divided into three sections: in the first part (**A**) socio-demographical and logistical information about the caregiver was collected, in the second part (**B**) caregivers were asked to assign a score for each item on a 4-point scale from 1 (lowest satisfaction) to 4 (highest satisfaction); in the third part (**C**), caregivers responded to five additional questions with yes/no answers.

Supplementary Figure 3 Adapted questionnaire based on the PedsQL 4.0 for toddlers (21), specifically tailored for 12-month-old children. The caregiver was asked to assign a score on a 5-point scale from 0 (never a problem) to 4 (almost always a problem) for each item assessing potential problems experienced by the child over the past month. The questionnaire was administered verbally by a healthcare provider from the IMDs RCC, with responses recorded by the operator.
